# The Antioxidant Machinery of Young and Senescent Human Umbilical Vein Endothelial Cells and Their Microvesicles

**DOI:** 10.1155/2017/7094781

**Published:** 2017-05-31

**Authors:** Guillermo Bodega, Matilde Alique, Lourdes Bohórquez, Sergio Ciordia, María C. Mena, Manuel R. Ramírez

**Affiliations:** ^1^Departamento de Biomedicina y Biotecnología, Universidad de Alcalá, 28871 Alcalá de Henares, Madrid, Spain; ^2^Departamento de Biología de Sistemas, Universidad de Alcalá, 28871 Alcalá de Henares, Madrid, Spain; ^3^Proteomics Facility, Centro Nacional de Biotecnología/CSIC, Campus de Cantoblanco, 28049 Madrid, Spain

## Abstract

We examine the antioxidant role of young and senescent human umbilical vein endothelial cells (HUVECs) and their microvesicles (MVs). Proteomic and Western blot studies have shown young HUVECs to have a complete and well-developed antioxidant system. Their MVs also contain antioxidant molecules, though of a smaller and more specific range, specialized in the degradation of hydrogen peroxide and the superoxide anion via the thioredoxin-peroxiredoxin system. Senescence was shown to be associated with a large increase in the size of the antioxidant machinery in both HUVECs and their MVs. These responses might help HUVECs and their MVs deal with the more oxidising conditions found in older cells. Functional analysis confirmed the antioxidant machinery of the MVs to be active and to increase in size with senescence. No glutathione or nonpeptide antioxidant (ascorbic acid and vitamin E) activity was detected in the MVs. Endothelial cells and MVs seem to adapt to higher ROS concentrations in senescence by increasing their antioxidant machinery, although this is not enough to recover completely from the senescence-induced ROS increase. Moreover, MVs could be involved in the regulation of the blood plasma redox status by functioning as ROS scavengers.

## 1. Introduction

Extracellular vesicles (EVs) are membranous vesicles released by different cell types into the blood, cerebrospinal and synovial fluids, urine, saliva, and bile. They are usually classified into three groups: microvesicles (MVs) (also known as ectosomes or microparticles), exosomes, and apoptotic bodies, depending on their size, morphology, density, and biogenetic origin [1]. Exosomes have an endosomal origin and are 50–100 nm across, MVs have a plasma membrane origin and are ≈1 *μ*m across, and apoptotic bodies are produced by apoptotic cells and are 1–5 *μ*m across [2]. Further information on the molecular contents and functions of EVs (intercellular communication, pathogenesis, cell adhesion, waste management, etc.) can be found in recent reviews [1, 3, 4].

**Figure 1:**
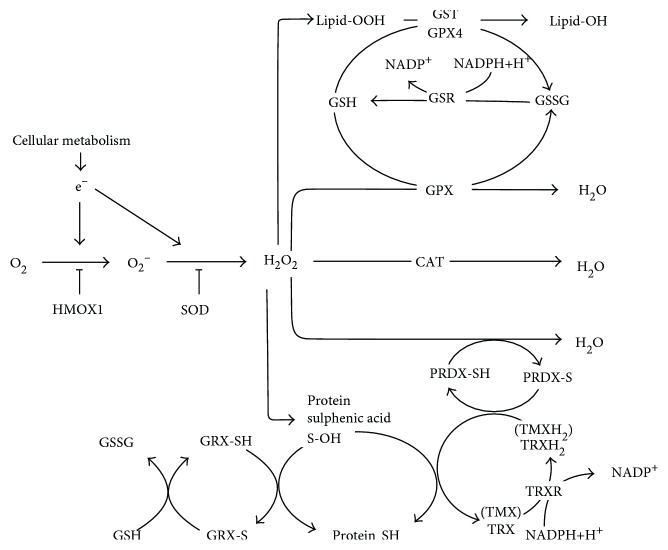
Diagram showing the main components of the peptide-involving antioxidant machinery. CAT: catalase; GPX: glutathione peroxidase; GRX: glutaredoxin; GSR: glutathione reductase; GST: glutathione S-transferase (K: kappa; O: omega; P: pi; T: theta); HMOX: heme oxygenase; mGST: microsomal glutathione S-transferase; PRDX: peroxiredoxin; SOD: superoxide dismutase; TMX: thioredoxin-related transmembrane protein; TRX: thioredoxin; TRXR: thioredoxin reductase. NADPH-synthesizing enzymes: G6PD: glucose-6-phosphate dehydrogenase; GLUD: glutamate dehydrogenase; IDH: isocitrate dehydrogenase; PGD: 6-phosphogluconate dehydrogenase.

Reactive oxygen species (ROS) are continuously produced by cells during normal metabolism but are also generated in large amounts when cells become stressed (e.g., when suffering hypoxia or starvation) or when an infection is present. They may therefore participate in healthy cell physiology as signalling molecules [5] and have an intracellular stress alarm function [6]. Unfortunately, their high reactivity can provoke unwanted changes in other important molecules, and cells have developed antioxidant mechanisms to protect themselves from such damage. The different components (enzymatic and nonenzymatic) of the cellular antioxidant machinery are discussed in reviews by Chaudière and Ferrari-Iliou [7] and Birben et al. [8].  Figure 1 explains the machinery involved in the antioxidant response, which is driven by the reducing power of NADPH [9].

Endothelial cells (simple squamous epithelium) form a lining on the inner surface of lymphatic and blood vessels (including the heart). While ROS play important physiological roles in vascular cells [10–12], it is well known that oxidative stress—a condition produced by an imbalance between oxidising products and antioxidant defences [13]—can alter many endothelial functions [14, 15], encouraging the appearance of atherosclerosis [16, 17], hypertension [18, 19], and cardiovascular diseases (CVD) [20–23].

Ageing is an important risk factor in the development of CVD [24]. Vascular ageing has been associated with the reduction of nitric oxide bioavailability, the dysfunction of endothelial progenitor cells, vascular inflammation, and the activation of a specific genetic program [25–28]. However, it also appears to be related to the senescence of the vascular endothelium [29]; oxidative stress has been related to both vascular ageing and endothelial dysfunction [30–32]. CVD and the senescence of vascular endothelial cells appear, therefore, to be intimately related.

The homeostatic maintenance of an appropriate blood redox status is essential; its imbalance has been reported to be involved in both CVD [19, 33, 34] and endothelial ageing [35]. Blood plasma receives ROS from different sites, including the cells of the tissue through which a vessel runs, from vascular muscle cells, endothelial cells, blood cells (especially erythrocytes), and even from within the plasma itself [36]. By virtue of their large overall mass (1 kg in a 70 kg person), and their direct contact with blood, endothelial cells are important regulators of blood redox status. The blood plasma, however, is also endowed with antioxidant mechanisms [37, 38].

MVs have been ascribed roles in coagulation, reticulocyte maturation, and angiogenesis (see reviews by Fleury et al. [39] and Yáñez-Mó et al. [1]) but may also help protect against oxidative stress. In this regard, treatments involving the administration of EVs have been reported to reduce oxidative stress in injured kidneys [40, 41] and in experimental colitis [42]. To further our knowledge of the antioxidant functions of endothelial cells and MVs, the present work examined the function of human umbilical vein endothelial cells (HUVECs) and HUVEC-derived MVs as ROS scavengers in the maintenance of redox homeostasis and the adaptability of these cells and ectosomes to senescence.

## 2. Materials and Methods

### 2.1. HUVEC Culture

Cryopreserved HUVECs (ATCC category number PCS-100-010) were cultured in endothelial growth medium (Lonza) supplemented with 10% heat-inactivated foetal bovine serum (Sigma-Aldrich). Cultures were maintained at 37°C in a 5% CO_2_ atmosphere at 95% humidity. The HUVECs were serially passaged (the replicative senescence model). Cells passaged <8 times (population doubling (PD) < 20; with PD calculated as [ln{number of cells harvested} − ln{number of cells seeded}/ln2]) were regarded as young endothelial cells, while those passaged 26–38 times (PD > 96) were regarded as senescent [43]. The proliferation rate of the latter cells is remarkably reduced, and more than 70% are positive for senescence-associated *β*-galactosidase. Prior to use, HUVEC extracts from cells passaged 4–8 (young pool) and from cells passaged 27–35 (senescent pool) were pooled (performed in triplicate).

### 2.2. Isolation and Characterization of Microvesicles

Young and senescent HUVEC-derived MVs were isolated from their culture medium. Briefly, samples were centrifuged using serial centrifugations (15 min at 3000 rpm, 30 min at 14,000 rpm), and pellets were frozen and stored at −20°C until their use. Prior to use, MVs from cells passaged 4–8 (young pool) and from cells passaged 27–35 (senescent pool) were pooled (performed in triplicate).

MVs from a medium containing young and senescent HUVEC cells were characterized in terms of size using a Beckman Coulter Cytomics FC500 flow cytometer running CXP software. MVs were understood to be those events gated with a size between 0.5 and 1.5 *μ*m; this gate was established from the side scatter versus forward scatter dot-plot produced in a standardization experiment using the SPHERO™ Flow Cytometry Nano Fluorescent Size Standard Kit (Spherotech). The latter has size-calibrated fluorescent beads ranging from 0.1 to 1.9 *μ*m in diameter. Events below 0.2 *μ*m were excluded in order to adequately distinguish true events from the background; events >1.9 *μ*m were excluded to prevent possible confusions with apoptotic bodies. The absolute number of MVs (events) per *μ*L was determined using Flow Count calibrator beads (Beckman Coulter) according to the manufacturer's recommendations and employing CXP software: (MVs counted × standard beads/L)/(standard beads counted). Data were recorded as the mean of three independent measurements of the same sample.

### 2.3. Proteomic Analysis

This analysis involved in-gel protein digestion followed by HPLC and mass spectrometry (MS). In order to obtain sufficiently large samples, the three HUVEC extract “young pools” (see Section 2.2) were mixed, as were the three HUVEC extract “senescent pools” and the corresponding pools for the MVs. These four samples were dissolved in lysis buffer (8 M urea, 2 M thiourea, 5% CHAPS, 2 mM TCEP-HCl, and protease inhibitors). Homogenization of the cells was achieved by ultrasonication (10 strokes, low amplitude) on ice. After homogenization, the lysed cells were centrifuged at 20,000 ×g for 10 min at 4°C, and the supernatant containing the solubilised proteins was used for LC-MS/MS experiment. Total protein concentration was determined using the Pierce 660 nm protein assay (Thermo). An aliquot of every sample was diluted with enough loading sample buffer and then applied onto 1.2 cm wide wells of a conventional SDS-PAGE gel (1 mm thick, 4% stacking, and 12% resolving). The run was stopped as soon as the front entered 1 cm into the resolving gel, so that the whole proteome became concentrated at the stacking/resolving gel interface. The unseparated protein bands were visualized by Coomassie staining, excised, cut into cubes (cross section 1 mm^2^), deposited in 96-well plates, and processed automatically in a Proteineer DP (Bruker Daltonics). The digestion protocol used was based on Shevchenko et al. [44] with minor variations: gel plugs were washed firstly with 50 mM ammonium bicarbonate and secondly with acetonitrile prior to reduction with 10 mM dithiothreitol in 25 mM ammonium bicarbonate solution; alkylation was performed with 55 mM indoleacetic acid in 50 mM ammonium bicarbonate solution. The gel pieces were then rinsed firstly with 50 mM ammonium bicarbonate and secondly with acetonitrile and then dried under a stream of nitrogen. Proteomics grade trypsin (Sigma-Aldrich) at a final concentration of 16 ng/*μ*L in 25% acetonitrile/50 mM ammonium bicarbonate solution was added and digestion allowed it to proceed at 37°C for 4 h. The reaction was stopped by adding 50% acetonitrile/0.5% trifluoroacetic for peptide extraction. The tryptic eluted peptides were dried by speed-vacuum centrifugation and then desalted onto StageTip C18 Pipette tips (Thermo Scientific) until examined by mass spectrometry.

A 1 *μ*g aliquot of each sample was subjected to 1D-nano LC ESI-MSMS analysis using an Eksigent Technologies nanoLC Ultra 1D plus nano liquid chromatography system coupled to high-speed TripleTOF 5600 mass spectrometer (SCIEX) with a Nanospray III source. The analytical column used was a silica-based reversed phase Acquity UPLC® M-Class Peptide BEH C18 Column (75 *μ*m × 150 mm, 1.7 *μ*m particle size, and 130 Å pore size) (Waters). The trap column was a C18 Acclaim PepMap™ 100 (Thermo Scientific) (100 *μ*m × 2 cm, 5 *μ*m particle diameter, 100 Å pore size), switched online with the analytical column. The loading pump delivered a solution of 0.1% formic acid in water at 2 *μ*L/min. The nanopump provided a flow rate of 250 nL/min and was operated under gradient elution conditions. Peptides were separated using a 2–90% mobile phase B gradient (mobile phase A: 2% acetonitrile, 0.1% formic acid; mobile phase B: 100% acetonitrile, 0.1% formic acid) for 250 min injection volume was 5 *μ*L.

Data acquisition was performed with a TripleTOF 5600 System (SCIEX) (ionspray voltage floating 2300 V, curtain gas 35 psi, interface heater temperature 150°C, ion source gas 1 25 psi, declustering potential 100 V). All data were acquired using information-dependent acquisition (IDA) mode with Analyst TF 1.7 software (SCIEX). The following IDA parameters were chosen: a 0.25 s MS survey scan in the mass range 350–1250 Da, followed by 35 MS/MS scans of 100 ms in the mass range 100–1800 (total cycle time: 4 s). Switching criteria were set to ions greater than a mass-to-charge ratio (m/z) of 350 and smaller than 1250, with a charge state of 2–5 and an abundance threshold of more than 90 counts/s (cps). Former target ions were excluded for 15 s. The IDA rolling collision energy (CE) parameters script was used for automatically controlling the CE.

MS and MS/MS data obtained for individual samples were processed using Analyst® TF 1.7 software. Raw data file conversion tools were used to generate mgf files which were then compared (using Mascot Server v.2.5.1 software; Matrix Science) to those in the UniProt *Homo sapiens* protein database. The latter contains 40,530 coding genes and their corresponding reversed entries. The search parameters were set as follows: carbamidomethyl (C) as the fixed modification and acetyl (protein N-term) and oxidation (M) as the variable modifications. Peptide mass tolerance was set to 25 ppm and 0.05 Da for fragment masses. Two missed cleavages were allowed. False discovery rates (≤1% at the spectral level) for peptide identification were calculated manually.

### 2.4. Western Blots

The total protein content of extracts from young and senescent HUVECs, and from their lysed MVs (performed using CytoBuster Protein Extraction Reagent lysis buffer (Millipore), which contains a protease and a phosphatase inhibitor cocktail (Roche)), was quantified using a BCA Protein Assay Kit (Pierce), employing BSA as the standard. Briefly, equal amounts of protein (10–50 *μ*g protein/lane) were diluted with a reducing sample buffer and separated by SDS/PAGE (10% gel) under reducing conditions. These proteins were then transferred onto nitrocellulose membranes (BioRad), blocked with TBS containing 0.1% Tween 20 and 5% dry nonfat milk for 1 h at room temperature (RT), and incubated in the same buffer with different primary antibodies (anticatalase, Abcam ab16731, dilution 1 : 1000, 60 kDa; anti-SOD1, Abcam ab16831, dilution 1 : 1000, 17 kDa; anti-SOD2, Abcam ab13533, dilution 1 : 1000, 25 kDa; anti-SOD3, Abcam ab171738, dilution 1 : 1000, 26 kDa; anti-TRX, Abcam ab26320, dilution 1 : 1000, 12 kDa). Anti-*β*-actin (Santa Cruz) (sc-47778, dilution 1 : 2000, 43 kDa) and anti-GAPDH (Millipore) (MAB374, dilution 1 : 2000, 38 kDa) were used as loading controls. After washing, the membranes were incubated with the appropriate Novex (Thermo Fisher) horseradish peroxidase-conjugated secondary antibodies (1 : 5000). Bands were visualized with Luminata Crescendo Western HRP substrate (Millipore). Protein quality and the efficacy of protein transfer were evaluated by red Ponceau staining. Bands were quantified using ImageJ software (NIH).

The 2-tailed Student *t*-test was used to analyse differences. Significance was set at *P* < 0.05.

### 2.5. Glutathione Determination by Specific Assay

The total glutathione content was measured using a specific assay kit (Cayman Chemical). One pool of young HUVECs (2 and 3 passages) and another of senescent HUVECs (29 and 30 passages) were sonicated in PBS, 1 mM EDTA, 7 pH, and centrifuged at 10,000 ×g for 15 min at 4°C. Their corresponding MVs (10^6^/mL) were sonicated in the same way. The supernatants of both centrifugations were processed following the kit instructions. Absorbance was measured using an ELX800 absorbance reader (BioTek). Absorbance data were normalized against the samples' protein concentration values. The experiment was repeated twice with three replicates per experiment. Data were recorded as means ± SD, and differences sought using the Student *t*-test.

### 2.6. Microanalysis: Scanning Electron Microscopy

One drop of suspension of MVs derived from young and senescent HUVECs (6000 MVs/mL) was placed on a clean, dry coverslip (12 mm diameter) with a water-repellent circle of approximately 6 mm diameter drawn using a pap pen, and incubated for 1 h in a humidified chamber at RT. After a brief wash in PBS, the MVs were fixed in 3% glutaraldehyde for 10 min, rinsed in buffer, dehydrated in an ethanol series, dried following the routine critical point drying procedure for scanning electron microscopy (SEM), and examined using a Hitachi TM-1000 SEM with an EDS system.

### 2.7. Functional Analysis

To examine the antioxidant activity of the MVs, HUVECs (young and senescent) were detached from their culture dishes and incubated with 5 *μ*M H2DCFDA (2′,7′-dichlorodihydrofluorescein diacetate) (Thermo Fisher) in PBS for 30 min at RT. Simultaneously, a solution of 50 *μ*M hydrogen peroxide in PBS was pretreated (or not (control)) with either young or senescent HUVEC-derived MVs (performed at RT for 30 min using 100,000 MVs/mL). HUVECs were centrifuged and washed to eliminate the H2DCFDA not incorporated into the cells. The cells were then immediately suspended in the hydrogen peroxide solution and seeded at 25,000 cells/well in black 96-multiwell plates. After 15 min incubation, a Victor X4 multilabel plate reader (Perkin Elmer) (excitation/emission 490 nm/535) was used to determine the emission of fluorescence by dichlorodihydrofluorescein (DCF) and to examine the product obtained after the oxidation of the H2DCFDA by the hydrogen peroxide. The fluorescence emission is directly proportional to the hydrogen peroxide content which, in turn, is inversely proportional to the antioxidant activity of the MVs.

The DPPH (1,1-diphenyl-2-picryl-hydrazyl) (Sigma-Aldrich) antioxidant assay [45] (slightly modified) was used to examine the possible antioxidant activity of the MVs' nonpeptide content. One millilitre of absolute ethanol, 500 *μ*L of 0.1 mM DPPH, and 500 *μ*L of sample, that is, 100,000 MVs/mL in water—sonicated or not—were placed in a reaction tube and incubated for 10 min at RT in the dark. The absorbance at 517 nm was then measured using an Implen NanoPhotometer (BioNova). The existence of molecules with antioxidant capacity leads to a loss in the violet colour depth of the DPPH (a consequence of the capture of an electron from the DPPH by the antioxidant molecules) and, consequently, a lower absorbance reading.

These experiments were repeated three times with four replicates per experiment. The results were analysed by three-way ANOVA (young and senescent cells, young and senescent MVs, and pretreatment or not). Data were recorded as means ± SD.

### 2.8. Mass Spectrometry

The glutathione, vitamin C, and vitamin E contents of the MVs were determined using an HPLC system coupled in-line to a TSQ Quantum triple quadrupole mass spectrometer (Thermo Scientific) equipped with an ESI source. Mass spectra were recorded in positive mode for glutathione and vitamin E and in negative mode for vitamin C. The stationary phase used in liquid chromatography was C18. Mass spectra of the column elutes were recorded in MS/MS mode using methanol and H_2_O as the mobile phase, adding 0.1% formic acid. Nitrogen was used as the ion source gas. The sheath gas flow rate was set at 40 (arbitrary units), the auxiliary gas flow rate at 1 (arbitrary units), and the ion sweep gas flow rate at 0.5 (arbitrary units). The capillary temperature was set at 350°C. Argon was used as the collision gas for collision-induced dissociation at a pressure of 1.5 mTorr (Q2). Data were acquired using Xcalibur Control Software.

The sought product ions for glutathione (M + H^+^ = 308.1 m/z) were (M + H)^+^ 231.06 m/z, (M + H)^+^ 275.57 m/z, and (M + H)^+^ 84.19 m/z. Those ions for vitamin E (M + H^+^ = 431.09 m/z) were (M + H)^+^ 165.09 m/z, (M + H)^+^ 137.13 m/z, and (M + H)^+^ 119.16 m/z, and for vitamin C (M-H^−^ = 175.1 m/z), they were (M-H)^−^ 87.20 m/z, (M-H)^−^ 115.12 m/z, and (M-H)^−^ 59.43 m/z.

All compounds were quantified using a calibration curve employing samples of known concentration. All determinations were performed in duplicate. Data processing was performed using LCquan 2.5 Control Software.

### 2.9. Immunofluorescence Microscopy

Young and senescent HUVECs were grown on 12 mm coverslips and incubated with DHE (dihydroethidium) for 30 min in a 5% CO_2_ atmosphere at 37°C. After washing in PBS (2 × 2 min), the cells were fixed for 12 min at RT in 4% paraformaldehyde diluted in PBS, washed (3 × 5 min), incubated in 100 mM glycine in PBS for 10 min at RT, and then incubated in 0.1% Triton X-100 (diluted in PBS) for 1 min at RT. After washing (2 × 5 min), the coverslips were incubated with Phalloidin-iFluor 1x (Abcam; ab176753) containing 1% BSA in PBS for 1 h at RT. After antibody incubation, the coverslips where washed (3 × 10 min) and mounted in 4 *μ*L drops of ProLong Diamond Antifade Mountant reagent (Invitrogen). Samples were allowed to rest at RT for at least 16 h before analysis using a fluorescence microscope and ImageJ software v.1.51 g. The Student *t*-test was used to analyse differences in the ROS content of the young and senescent cells. Significance was set at *P* < 0.05.

## 3. Results

### 3.1. Proteomic and Molecular Analysis

Proteomic analysis detected a large number of proteins (≈3800) in the HUVECs and MVs taken together, but only 28 were found to be closely related to antioxidant metabolism (Table 1).

HUVECs were found to have a complete antioxidant machinery that strongly increased in size with age. Increases were seen in the recorded values for the PS, PSM and NP (see the Figure 1 legend). Three new proteins (GSTT1, GSTT2, and TMX2; protein acronyms are explained in the Figure 1 legend) were detected in the senescent cells. In addition, 11 proteins expressed in the senescent HUVECs, including CAT, SOD2, and the glutathione-related proteins GPX, GRX, GSR, and GST, showed a >25% increase in abundance compared to those in young HUVECs.

Proteomic analysis showed the MVs to be less rich in antioxidant proteins than the HUVECs; only 8 proteins were detected in young MVs compared to 28 in young HUVECs, rising to 10 in senescent MVs compared to 31 in senescent HUVECs. The two new proteins detected in the senescent MVs were CAT and SOD2. In addition, the PS, PSM and NP values were, in general, increased in senescent MVs. Table 1 clearly shows the proteomic content of the MVs to be very different to that of the HUVECs. Indeed, the antioxidant proteomic content of the MVs mainly involves the thioredoxin-peroxiredoxin (TRX-PRDX) system, while the HUVEC system is much more complete.

The expression of five enzymes capable of synthesizing NADPH (the fuel of the antioxidant machinery), that is, G6PD, IDH1 and IDH2, MDH, and PGD (bottom of Table 1), is of particular interest. The PS, PSM, and NP data for these enzymes were similar for both the young and senescent HUVECs; however, they were different for the young and senescent MVs. The senescent MVs contained a new protein, G6PD, and showed a higher PS for PGD and GLUD1. GLUD1 was the most abundant protein in MVs of both ages.

**Figure 2:**
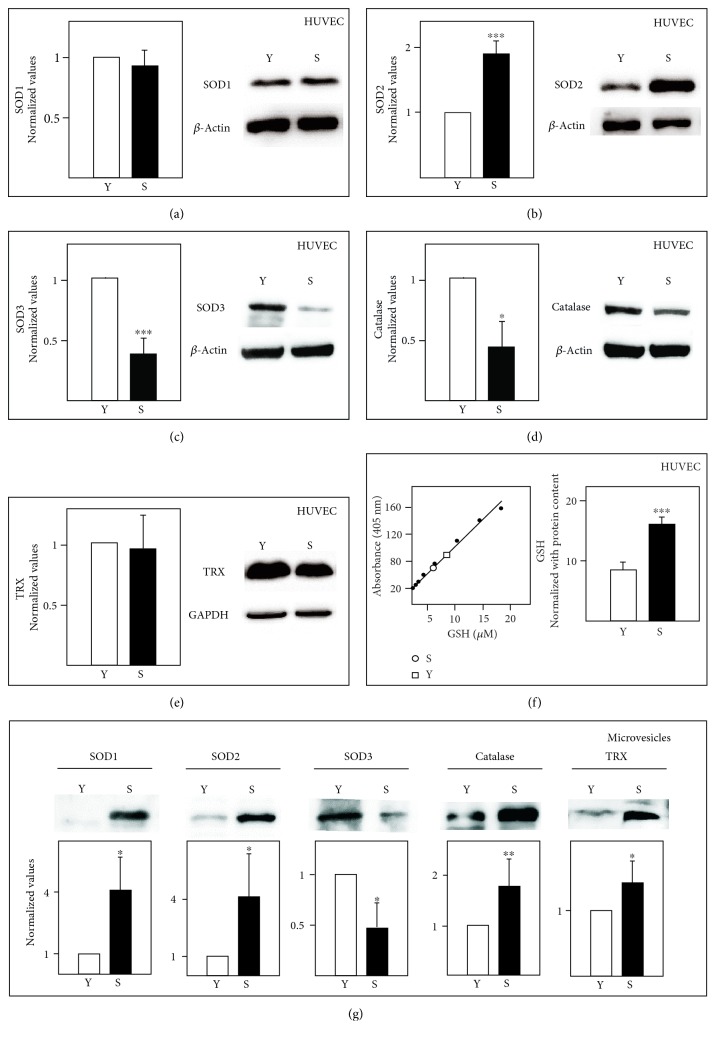
Western blot analysis of SOD1, SOD2, SOD3, catalase, and TRX in HUVECs (a–e) and MVs (g). GSH (total content) was analysed using a specific kit (f); the left panel shows the standard curve and the right panel the total GSH content. Y: young; S: senescent. HUVEC protein data were normalized against *β*-actin (a–d) or GAPDH (e); however, the GSH data (f) were normalized against the protein content of the sample. Protein data for the MVs was normalized against the intensity of red Ponceau staining. Bars represent mean ± SD (*n* = 3 pools). ^∗^*p* < 0.05, ^∗∗^*p* < 0.01, ^∗∗∗^*p* < 0.001.

More accurate determinations of the two most important antioxidant enzymes (CAT and SODs) and metabolites (GSH and TRX) were also performed in both HUVECs and MVs (Figure 2). CAT, SODs, and TRX levels were determined by Western blot analysis, and GSH (given its small size) using a specific kit and mass spectrometry. In the HUVECs, and at both cell ages, SOD1 (Cu-Zn-SOD, cytosolic) and SOD2 (Mn-SOD, mitochondrial) expressions were found to be similar by both proteomic and Western blot analysis. SOD3, the extracellular isoform, which was not detected in proteomic analysis, was detected in Western blot analysis and showed a significant reduction in concentration in senescent compared to young HUVECs. An unexpected and significant reduction was also observed for CAT in senescent cells. No difference was observed in the TRX content of young and senescent HUVECs, in agreement with the results of the proteomic analysis.

Western blotting also showed the CAT, SOD1, SOD2, and TRX contents to be significantly increased in senescent MVs compared to those in young MVs; in contrast, a significant reduction was seen in the SOD3 content. GSH was more abundant in senescent than in young HUVECs. This protein could not be detected in the MVs using either the GSH kit assay or mass spectrometry methods.

**Figure 3:**
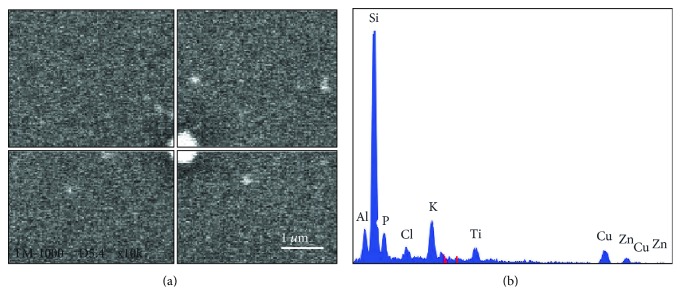
Microanalysis image of a young MV analysed in spot mode (a). Microanalysis plot of this spot (b). Si, Al, and Ti are components of the coverslip glass.

Since SODs contain Cu, Zn, Mn, and Fe, the location of these proteins in the MVs was studied using a microanalysis scanning electron microscope. Almost all the MVs thus studied showed the presence of Cu and Zn (Figure 3).

### 3.2. Functional Analysis

Three different assays were used in the functional analysis: (1) the DHE assay, to detect superoxide anion in HUVECs, (2) DCF assay, to analyse the activity of the MVs as ROS scavengers in the extracellular media, and (3) the DPPH assay, to test the content of nonpeptide antioxidant molecules in MVs.

**Figure 4:**
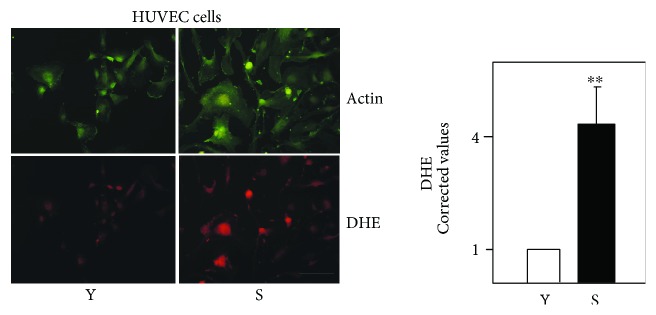
DHE analysis of HUVECs. Actin is labelled with a green fluorophore (upper images), DHE emits red fluorescence (lower images). Y: young cells; S: senescent cells. The images shown are representative of 60 for each cell age. Scale bar: 100 *μ*m. ^∗∗^*p* < 0.05.

The oxidative activity of young and senescent HUVECs was first examined using the DHE test, which detects the superoxide anion. The senescent cells showed a significantly stronger DHE signal and, consequently, a greater production of the superoxide anion than did the young cells. The senescent cells were also larger in size (Figure 4).

**Figure 5:**
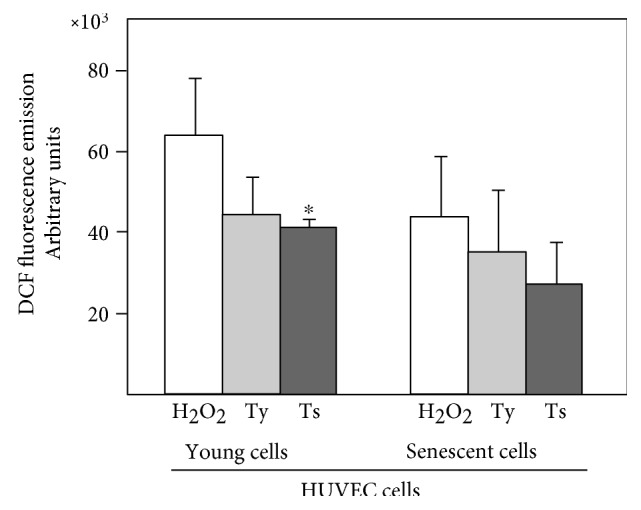
Fluorescence emission by DCF. The culture medium included 50 mM of hydrogen peroxide. The bars reveal the effect of pretreating the medium with young MVs (Ty) or senescent MVs (Ts). ^∗^*p* < 0.05, Ts versus H_2_O_2_ in young cells.

The antioxidant activity of young and senescent MVs was studied via the fluorescence emission of DCF, a product obtained after the oxidation of H2DCFDA by—in the present test—hydrogen peroxide. Briefly, HUVECs were treated with H2DCFDA, which penetrates them. The cells were then placed in a culture medium including hydrogen peroxide previously conditioned—or not—by incubating with young or senescent MVs. The antioxidant effect of the MVs was then quantified by analysing the fluorescence emission of the DCF in the HUVECs. The greater the antioxidant effect of the MVs, the fewer hydrogen peroxide molecules in the medium culture, leading to a smaller amount of DCF and a decay in fluorescence. Pretreatment with MVs always induced a reduction in fluorescence (Figure 5); note that pretreatment with senescent MVs (Ts) always induced a stronger reduction than pretreatment with young MVs (Ty). Moreover, the senescent cells always showed a smaller fluorescence emission than younger cells. A strong, inverse correlation (*r* = −0.95) was detected between the antioxidant content (PS data, taking together the PS of the cells plus the PS of the MVs in the MV pretreatment scenario) and fluorescence emission values.

**Figure 6:**
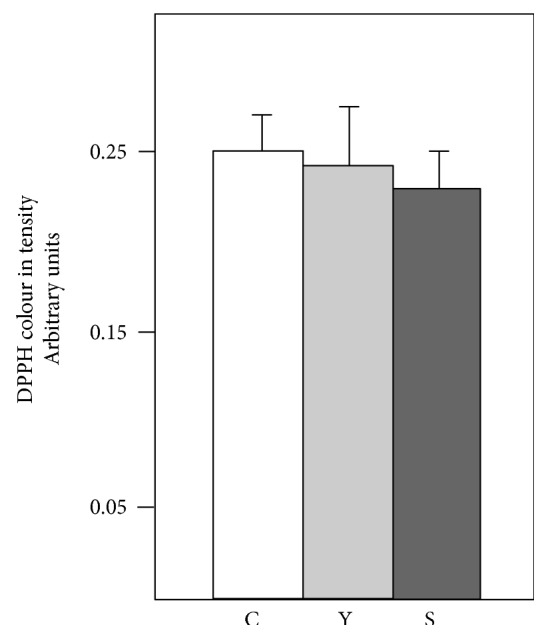
DPPH test. The colour intensity of the solution was quantified before (C: control) and after the addition of young (Y) or senescent (S) MVs.

The possibility that MVs contain nonpeptide antioxidant molecules was tested using the DPPH colorimetric test. This molecule loses its violet colour in the presence of antioxidant molecules. As a control, samples with different concentrations of ascorbic acid were prepared, and a concentration-dependent loss of colour was observed (data not included). However, neither the young nor the senescent MVs had much effect on the colour of the DPPH solution (2.71 and 5.52% reduction, resp.) (Figure 6). Mass spectrometry analysis was performed to detect the presence of ascorbic acid and vitamin E in the extracts of young and senescent MVs, but these molecules were not found.

## 4. Discussion

The present work shows that HUVEC-derived MVs contain active antioxidant molecules and have an important antioxidant role. A number of functions have been ascribed to MVs [1, 3, 4], but this is the first time that their antioxidant activity has been clearly demonstrated. It would appear to provide an important aid in maintaining the blood redox status, and thus help in preventing the different CVDs associated with the latter's imbalance [19, 33, 34].

MVs from blood have been subjected to proteomic analysis by other authors (see a review by Tissot [46]). Some detected no antioxidant proteins [47, 48] while others reported them to contain GPX, GST, PRDX1–3, and SOD2 [49]; GPX, PRDX5, TRX, and SOD2 [50]; GST, SOD2, and PRDX6 [51]; and GPX3, PRX1, PRX2, and PRX4 [52]. CAT and the three SOD isoforms have also been reported to be present and functional in HUVEC MVs [53]. SOD2, TRX, TRXR, and CAT have also been detected in the MVs of *Cryptococcus neoformans*, a unicellular eukaryote parasite [54]. As shown in Table 1, all these proteins (and others) were detected in the present MVs.

Some differences were detected when comparing the data of Western blot and proteomic analysis. SOD3 was only detected by Western blot; SOD1 and TRX were detected in MVs analysed by Western blot but not by proteomic analysis. This might be attributed to the higher sensitivity of the Western blot analysis, as suggested by the data obtained for catalase in MVs: detection in young and senescent MVs by Western blot but only in senescent MVs by proteomic analysis. Furthermore, proteomic analysis demonstrated an increase whereas Western blot analysis demonstrated a decrease in catalase content in senescent cells. Because of the different preparation of the samples in proteomic and Western blot analysis, the changes in catalase induced by senescence that could affect its solubility might explain the decrease observed in Western blot.

Plasma redox levels are carefully regulated and maintained at a low level [55]. The main cells involved are endothelial cells, although the blood also possesses a complete array of antioxidants, including ascorbic acid, urate, *α*-tocopherol, and different proteins with sulphydryl groups [37, 38]. The present proteomic results show that HUVECs possess a complete and abundant antioxidant machinery, though perhaps less well developed than that of renal epithelium [56]. The present results also show HUVEC-derived MVs to contain components of these antioxidant systems that might help endothelial cells in their regulation of blood plasma redox status. A functional role as protectors against oxidative stress was suggested by Soleti et al. [53]. Later, experimental treatments were devised using EVs to reduce oxidative stress in the injured kidney [40, 41] and in experimental colitis [42]. The possibility that MVs act as scavengers of nitric oxide and other free radicals, or that they carry signalling molecules related to redox-regulated processes, has also been proposed [57]. A different role has been suggested for the antioxidant activity detected in the parasite cited above; the antioxidant components may serve to eliminate the oxidant molecules produced by the host, making the parasite more virulent [54].

The present results demonstrate that MVs function as antioxidant elements, mainly in the destruction of the main ROS (i.e., hydrogen peroxide and the superoxide anion). Note that they contain only molecules (see Figure 1) that act directly on hydrogen peroxide (GPX, CAT, and the TRX-PRDX system) and the superoxide anion (SODs); molecules that act on proteins and lipids are not present. The present results suggest that neither ascorbic acid nor vitamin E is present in the MVs studied, although this might be expected (these types of molecule are usually found in plasma [37, 38]). Thus, this antioxidant system does not seem to be based at all on GSH, but rather on the TRX-PRDX system. All in all, the ensemble of antioxidant enzymes capable of synthesizing NADP might give autonomy to MVs for ROS scavenging.

In our functional experiment (DCF assay), MVs do not seem to have a very strong effect as ROS scavengers, in spite of the strong immunoreactive signals of their antioxidant enzymes. One plausible explanation is that enzymatic activity might be affected by the process of isolation and conservation of MVs, as well as by the conditions of the functional analysis; moreover, a high amount of protein was loaded into the gel wells in order to facilitate the detection of these enzymes.

Irrespective of any antioxidant scavenger function, it has recently been suggested that MVs produce ROS as part of signalling processes in endothelial cells [58]. Thus, MVs might act as both ROS scavengers and ROS synthesizers. This paradox might be explained in that MVs appear to be a heterogeneous population—as suggested, for example, by their wide variation in size. The same has been suggested for exosomes [59]. It should also be noted, however, that MVs have a highly specific antioxidant machinery: it is not simply a reduced version of that shown by endothelial cells but a selective reduction that suggests MVs carry a specific cargo. Certainly, specific mechanisms have been suggested for loading MVs with proteins [60], lipids [61], and RNA [62].

Antioxidant molecules in the endothelium have been reported in previous proteomic studies, although only a very small number were ever identified [63, 64]. High antioxidant activity has also been suggested for endothelial progenitor cells [65], and large contents of peroxide detoxifying enzymes were demonstrated at the blood-brain barrier long ago [66].

Vascular ageing is a consequence of redox imbalances [31]. However, it is also well known that ageing per se increases the formation of ROS in endothelial cells [24, 25, 30, 67]. Alterations in the concentrations of NADPH oxidases and other oxidases have been implicated as the source of this excess endothelial ROS [68, 69]. It is also accepted that ROS-induced vascular ageing is mediated by a reduction in the bioavailability of nitric oxide [14, 15, 24, 25, 29, 32]. Moreover, ROS may affect the integrity of the telomeres [70].

The present results clearly show not only a higher superoxide anion production (DHE assay) but also a higher ROS scavenger activity (DCF assay) as well and a large increase in the size of the antioxidant machinery in both senescent HUVECs and senescent MVs. These paradoxical results might be explained as an adaptive response designed to buffer the greater production of ROS in these senescent structures. However, it would seem that this mechanism is unable to fully maintain a balanced redox status.

Finally, just as nucleus-free red blood cells are structures with the function of oxygen transport, or platelets are mainly involved in coagulation, MVs (or at least a subpopulation of these) might be considered structures with the function of scavenging ROS. The antioxidant machinery of the MVs is exclusively involved in ROS scavenging and not in the recovery of the MVs' own peroxidised lipids and proteins, which probably leads to their ageing and their final degradation.

## 5. Conclusions

This study shows that HUVECs have a complete and well-developed antioxidant machinery. HUVEC-derived MVs also have functional peptide antioxidant components that allow them to act as ROS scavengers. The antioxidant complement of MVs is not a scaled-down version of the HUVEC machinery but a specific complement involved only in hydrogen peroxide and superoxide anion degradation via the TRX-PRDX system. HUVEC-derived MVs possess not only antioxidant enzymes and antioxidant peptides but also NADPH-synthesizing enzymes, which could provide them with a degree of autonomous antioxidant activity. Finally, as MVs and HUVECs age—a condition involving growing oxidative stress—their antioxidant machinery is potentiated.

## Figures and Tables

**Table 1:** Proteomic analysis of young and senescent HUVECs and young and senescent MVs. PS: protein score; PSM: peptide-spectrum match; NP: number of peptides (MS/MS scores are sums for the validated peptides assigned to each protein); C: coverage. PS, PSM, and NP are usually considered quantitative variables in proteomic analysis. Bold numbers in the “senescent columns” indicate a 25% increase; bold numbers in the “young columns” indicate a 25% reduction. The five proteins at the bottom of the table are the enzymes that synthesize NADPH. The acronyms key is included in the Figure 1 legend.

UniProt		Young HUVECs				Senescent HUVEC				Young MVs				Senescent MVs			
		PS	PSM	NP	C %	PS	PSM	NP	C %	PS	PSM	NP	C %	PS	PSM	NP	C %
P04040	**CAT**	171.1	3.0	3.0	15.9	**345.1**	**7.0**	**7.0**	27.1					**146.4**	**3.0**	**3.0**	9.5
P07203	**GPX1**	334.1	7.0	6.0	43.8	376.4	**9.0**	6.0	43.8	**172.6**	**3.0**	**3.0**	35.5	38.2	1.0	1.0	6.9
P22352	**GPX3**					**36.8**	**1.0**	**1.0**	5.3								
P36969	**GPX4**	48.8	1.0	1.0	19.3	**68.5**	**4.0**	**4.0**	22.8								
P35754	**GRX1**	36.2	1.0	1.0	10.4	**54.5**	1.0	1.0	11.3								
O76003	**GRX3**	293.3	7.0	7.0	42.1	**469.8**	**9.0**	8.0	47.5								
P00390	**GSR**	199.0	5.0	4.0	23.4	**356.3**	**7.0**	**7.0**	29.9								
Q9Y2Q3	**GSTK1**	377.5	5.0	5.0	39.8	372.0	6.0	6.0	41.2								
P78417	**GSTO1**	579.1	17.0	11.0	44.4	**770.8**	**25.0**	**14.0**	44.4								
P09211	**GSTP1**	871.0	29.0	14.0	65.2	1068.5	**37.0**	14.0	65.2								
P30711	**GSTT1**					**31.7**	**1.0**	**1.0**	5.4								
P0CG29	**GSTT2**					**261.9**	**7.0**	**5.0**	29.9								

P09601	**HMOX1**	533.3	12.0	9.0	43.1	**813.2**	**16.0**	**15.0**	58.0								
Q99735	**mGST2**	**89.9**	1.0	1.0	13.6	43.2	1.0	1.0	13.6								
O14880	**mGST3**	32.2	1.0	1.0	8.6	**336.1**	**6.0**	**5.0**	34.9								
Q06830	**PRDX1**	709.3	29.0	14.0	52.3	688.1	30.0	13.0	50.3	441.3	18.0	9.0	47.2	418.5	16.0	8.0	58.3
P32119	**PRDX2**	359.3	17.0	7.0	40.9	**507.4**	16.0	**10.0**	37.4	253.4	9.0	4.0	32.8	266.3	10.0	4.0	23.7
P30048	**PRDX3**	**436.2**	**15.0**	8.0	31.3	316.7	11.0	7.0	34.8	**191.8**	**4.0**	**3.0**	20.3	92.3	2.0	2.0	25.8
P30044	**PRDX4**	401.1	11.0	7.0	39.1	366.6	11.0	6.0	25.5	**443.0**	**14.0**	**8.0**	45.8	212.0	7.0	4.0	17.3

Q13162	**PRDX5**	479.4	14.0	9.0	42.5	503.7	14.0	8.0	37.4	39.2	1.0	1.0	27.1	**317.8**	**10.0**	**6.0**	39.7
P30041	**PRDX6**	891.1	21.0	15.0	68.8	1053.9	**40.0**	17.0	68.8	119.5	2.0	2.0	25.0	**349.5**	**9.0**	**6.0**	47.8
P00441	**SOD1**	80.4	1.0	1.0	14.9	71.4	**2.0**	1.0	15.6								
P04179	**SOD2**	156.3	4.0	3.0	19.4	**523.1**	**12.0**	**9.0**	38.3					**144.0**	**3.0**	**3.0**	33.8
Q9H3N1	**TMX1**	137.5	3.0	2.0	15.0	**178.4**	**4.0**	**3.0**	15.0								
Q9Y320	**TMX2**					**78.2**	**2.0**	**2.0**	8.4								
Q9H1E5	**TMX4**	73.4	2.0	**2.0**	7.2	61.2	2.0	1.0	3.7								
P10599	**TRX**	285.4	**8.0**	5.0	34.3	264.7	5.0	4.0	34.3								
Q16881	**TRXR1**	1015.5	22.0	18.0	39.3	**1274.0**	24.0	18.0	37.6	154.6	2.0	2.0	7.2	138.7	**3.0**	2.0	14.0

P11413	**G6PD**	878.5	28.0	20.0	54.4	950.8	24.0	20.0	51.3					**45.0**	**1.0**	**1.0**	11.8
P52209	**PGD**	1072.8	26.0	18.0	48.7	877.0	27.0	15.0	41.6	161.6	3.0	3.0	22.2	160.0	3.0	3.0	24.2
P00367	**GLUD1**	989.7	28.0	19.0	40.0	**1274.2**	31.0	20.0	48.9	329.0	11.0	5.0	24.0	**637.0**	**17.0**	**11.0**	35.5
O85874	**IDH1**	738.9	16.0	13.0	44.2	908.8	19.0	16.0	49.3								
P48735	**IDH2**	**681.6**	**16.0**	**14.0**	34.5	490.0	10.0	9.0	35.4	**254.0**	**6.0**	**5.0**	38.7	133.0	3.0	3.0	30.8
